# Arylsulfonylamino-Benzanilides as Inhibitors of the Apical Sodium-Dependent Bile Salt Transporter (SLC10A2)

**DOI:** 10.3390/molecules18066883

**Published:** 2013-06-10

**Authors:** Hong-Tao Liu, Hong-Wei He, Xiao-Guang Bai, Ju-Xian Wang, Chang-Liang Xu, Shi-Ying Cai, Rong-Guang Shao, Yu-Cheng Wang

**Affiliations:** 1 Institute of Medicinal Biotechnology, Chinese Academy of Medical Sciences and Peking Union Medical College, Beijing 100050, China; 2 Department of Internal Medicine and Liver Center, School of Medicine, Yale University, New Haven, CT 06520, USA

**Keywords:** ASBT inhibitors, bile acids, arylsulfonylaminobenzanilides, cholesterol lowering drug

## Abstract

The apical sodium-dependent bile salt transporter (ASBT) plays a pivotal role in maintaining bile acid homeostasis. Inhibition of ASBT would reduce bile acid pool size and lower cholesterol levels. In this report, a series of novel arylsulfonylaminobenzanilides were designed and synthesized as potential inhibitors of ASBT. Most of them demonstrated great potency against ASBT’s bile acid transport activity. In particular, compound **5g_2_** inhibited ASBT activity with an IC_50_ value of 0.11 μM. These compounds represent potential cholesterol-lowering drugs.

## 1. Introduction

Coronary artery disease (CAD) is a leading cause of death around the World [1]. High levels of cholesterol are one of the main causes of CAD, which greatly increases the risk of formation of plaques and atherosclerosis [2]. As a result, lowering cholesterol is beneficial to the prevention of CAD. Bile acids are metabolites of cholesterol. Bile acids are synthesized in the liver and released into the duodenum after a meal to facilitate lipid absorption. In the ileum, most bile acids are reabsorbed by the apical sodium-dependent bile salt transporter (ASBT, SLC10A2) and transported back to the liver through enterohepatic circulation. However, there is a fraction of bile acids that escape intestinal reabsorption, and are excreted with feces. The loss of bile acid in feces triggers *de novo* bile acids synthesis from cholesterol in order to maintain the bile acid pool size. This process represents the major route for the elimination of cholesterol from the body [3].

The argument that the increase of bile acids excretion can reduce hepatic and serum cholesterol was proven by the usage of bile acid sequestrants (BASs) [4]. As one of the most commonly used drugs for treating hypercholesterolemia and hyperlipidemia, BASs bind to bile acids and prevent their re-absorption in the intestine. Although BASs have a good safety record and synergistic effects when combined with statins, they still suffer from poor patient compliance due to their high dosages and bad palatability [5]. Therefore, the development of new drugs with similar physiological response to BASs, but with improved palatability, is in demand for lowering cholesterol.

**Figure 1:**
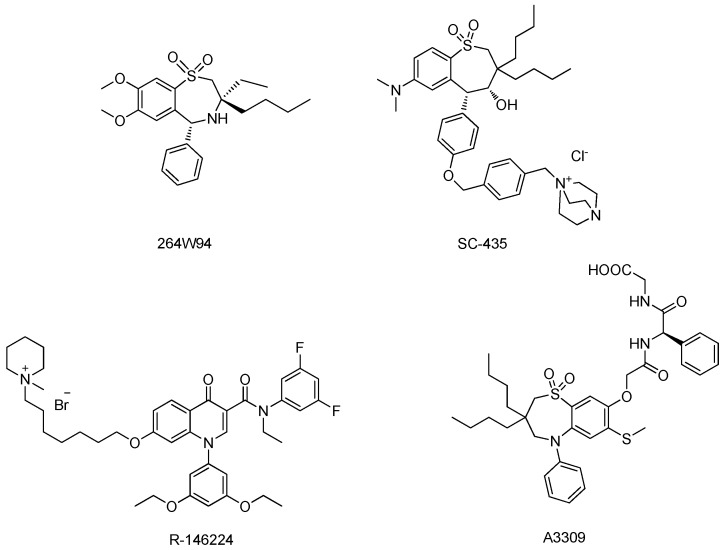
Structures of ASBT inhibitors.

ASBT plays a critical role in maintaining the bile acids pool size by reabsorbing bile acids in the ileum [6,7,8]. Ablation of ASBT function reduces bile acid pool size in mouse. Lower serum cholesterol levels were also observed in humans with ASBT mutations [9]. Therefore, ASBT is an attractive target for developing new cholesterol-lowering drugs [10]. Inhibition of ASBT function can increase bile acid fecal loss, which in turn stimulates hepatic conversion of cholesterol into bile acids [11]. Because ASBT is localized on the apical membrane of the lumen in the ileum, its inhibitors can block ASBT activity without entering the circulation system. This non-systemic character of ASBT inhibitors implies a low risk of potential systemic toxicity and drug–drug interactions [12,13]. So far, a number of ASBT inhibitors having various structural characteristics have been synthesized. Among of them, three candidates—264W94, SC-435 and R-146224 (Figure 1) were reported to block bile acid re-absorption and reduce cholesterol levels significantly in animal models [14,15,16]. In addition, it has recently been demonstrated in a Phase Ⅲ trial that A3309 (Figure 1), another ASBT inhibitor, can be used to treat patients with chronic idiopathic constipation (CIC).

**Figure 2:**
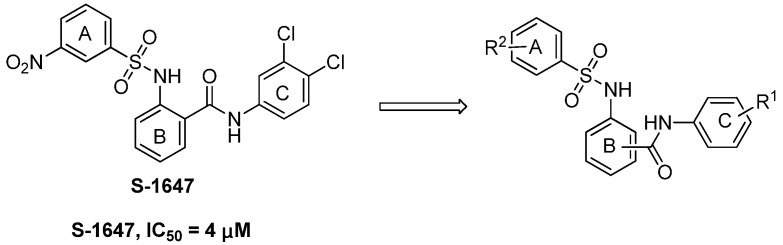
Design of arylsulfonylaminobenzanilides.

Baringhaus *et al.* developed a reliable 3D QSAR pharmacophore model for ASBT and screened a novel compound S-1647 (Figure 2) with considerable inhibition against ASBT (IC_50_: 4 μM) [17]. The simpler structure of S-1647 containing the three benzene rings A, B and C, compared with 264W94, SC-435 and R-146224, attracted our attention. We decided to make structural modifications on S-1647. In this study structure–activity relationships (SAR) of the relative positions of the ring C carbamyl group to ring B were investigated first, leading to three classes of compounds, and then various substitutions of rings A and C were added (Figure 2). Our primary objective was to optimize the potency of S-1647 against ASBT and a preliminary SAR was also explored to facilitate the further study of this class of compounds.

## 2. Result and Discussion

### 2.1. Chemistry

**Scheme 1:**
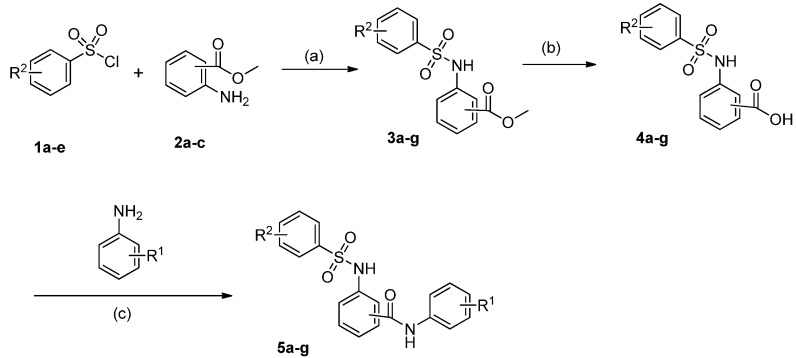
The synthesis of arylsulfonylamino-benzanilides **5a**–**g**.

The synthetic pathways to this series of target compounds were shown in Scheme 1. Nucleophilic substitution of substituted sulfonyl chlorides **1a**–**e** with various aminobenzoates **2a**–**c** in the presence of pyridine in tetrahydrofuran (THF) gave arylsulfonylaminobenzoates **3a**–**g**. Hydrolysis of the benzoates **3a**–**g** in a NaOH-H_2_O-EtOH system yielded the corresponding arylsulfonylaminobenzoic acids **4a**–**g**. Coupling of the benzoic acids **4a**–**g** with commercially available substituted anilines in the presence of 1-hydroxybenzotrizole (HOBt), 1-ethyl-3-(3-dimethylaminopropyl)carbodiimide hydro- chloride (EDC**^.^**HCl) and ethyldiisopropylamine (DIEA) in dimethylformamide (DMF) afforded the target compounds **5a**–**g**.

*Reagents and conditions*: (a) Pyridine, THF, r. t. (b) NaOH, EtOH, reflux; (c) selected amine, HOBt, EDC HCl, DIEA, DMF, r.t.

### 2.2. Biological Activity

*In vitro* inhibitory activity of all target compounds against ASBT was evaluated using a radioisotope-based assay. All the newly synthesized derivatives were initially tested at 10 μM concentration (Table 1).

**Table 1:** The structures and ASBT inhibitory rate of **5a_1_**–**a_4_**, **5b_1_**–**b_3_** and **5c_1_**–**c_2_**. 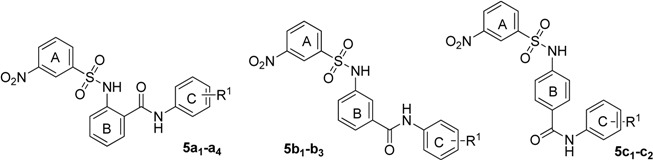

**Compd.**	**R^1^**	**Inhibition (%) ^a^**	**Compd.**	**R^1^**	**Inhibition (%) ^a^**
**5a_1_** (S-1647)	3,4-dichloro	79.3 ± 5.2	**5b_2_**	3-difluoromethoxy	30.6 ± 4.3
**5a_2_**	3-chloro-4-fluoro	95.4 ± 3.3	**5b_3_**	3,4-dichloro	62.4 ± 2.9
**5a_3_**	3-difluoromethoxy	89.0 ± 2.6	**5c_1_**	3- trifluoromethoxy	29.6 ± 3.5
**5a_4_**	3-trifluoromethoxy	83.8 ± 3.4	**5c_2_**	3,4-dichloro	34.8 ± 3.7
**5b_1_**	3-chloro-4-fluoro	66.3 ± 5.0			

^a^ Values represent the percent inhibition of ASBT at 10 µM of the test compounds and are the average of three independent experiments.

The results suggest that the activity against ASBT decreased while the relative distance of the ring C carbamyl group to ring B increased. For example, *ortho* position compounds **5a_1_**–**a_4_** exhibited better activity than the corresponding *meta* position compounds **5b_1_**–**b_3_** and *para* position compounds **5c_1_**–**c_2_**, so the carbamyl group in the *ortho* position with respect to the ring B is preferably for activity. Then, we explored the nitro group position in the ring A, and prepared two types of compounds (Table 2).

**Table 2:** The structures and ASBT inhibitory rate of **5a_5_**–**a_10_** and **5d_1_**–**d_6_**. 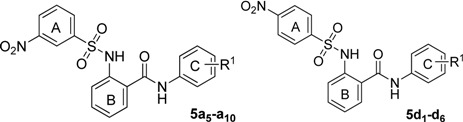

**Compd.**	**R^1^**	**Inhibition (%) ^a^**	**Compd.**	**R^1^**	**Inhibition (%) ^a^**
**5a_5_**	3,5-difluoro	21.6 ± 4.1	**5d_1_**	2,4- dichloro	66.9 ± 3.5
**5a_6_**	3-hydroxy-4-methoxy	32.1 ± 3.3	**5d_2_**	3-chloro-4-fluoro	75.5 ± 4.4
**5a_7_**	3,5-dichloro	56.3 ± 2.8	**5d_3_**	3-trifluoromethyl-4-methyl	73.7 ± 3.8
**5a_8_**	2,4-dichloro	99.1 ± 2.3	**5d_4_**	3-difluoromethoxy	58.8 ± 4.3
**5a_9_**	3- chloro	46.9 ± 3.6	**5d_5_**	3- trifluoromethoxy	66.7 ± 3.9
**5a_10_**	3-trifluoromethyl-4-methyl	90.7 ± 4.2	**5d_6_**	3,4- dichloro	73.9 ± 2.2

^a^ Values represent the percent inhibition of ASBT at 10 µM of the test compounds and are the average of three independent experiments.

The position of the nitro group was considered an important factor in the activity. Apparently, compounds **5a_1_**–**a_4_**, **5a_8_**, and **5a_10_** with 3-nitro groups showed better activity than the corresponding 4-nitro analogues **5d_1_**–**d_6_**, respectively. Finally, we investigated the R^2^ group in ring A, and designed three types of compounds (Table 3).

**Table 3:** The structures and ASBT inhibitory rate of **5e_1_**–**e_5_**, **5f_1_**–**f_5_** and **5g_1_**–**g_5_**. 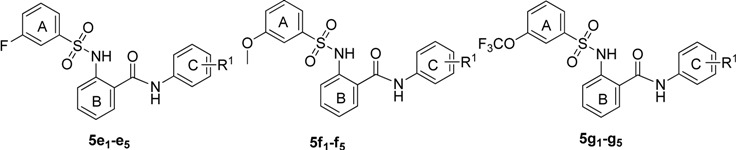

**Compd.**	**R^1^**	**Inhibition (%) ^a^**	**Compd.**	**R^1^**	**Inhibition (%) ^a^**
**5e_1_**	2,4-dichloro	42.6 ± 3.4	**5f_4_**	3-trifluoromethoxy	25.8 ± 2.1
**5e_2_**	3-chloro-4-fluoro	63.2 ± 3.8	**5f_5_**	3,4-dichloro	20.1 ± 1.9
**5e_3_**	3-trifluoromethyl-4-methyl	57.6 ± 2.9	**5g_1_**	2,4-dichloro	89.3 ± 3.3
**5e_4_**	3-trifluoromethoxy	45.7 ± 3.6	**5g_2_**	3-chloro-4-fluoro	98.6 ± 2.6
**5e_5_**	3,4-dichloro	40.6 ± 2.8	**5g_3_**	3-trifluoromethyl-4-methyl	91.8 ± 3.5
**5f_1_**	2,4-dichloro	21.2 ± 3.0	**5g_4_**	3-trifluoromethoxy	88.1 ± 2.7
**5f_2_**	3-chloro-4-fluoro	59.5 ± 3.6	**5g_5_**	3,4-dichloro	80.8 ± 4.3
**5f_3_**	3-trifluoromethyl-4-methyl	43.0 ± 3.2			

^a^ Values represent the percent inhibition of ASBT at 10 µM of the test compounds and are the average of three independent experiments.

The electronic properties of the R^2^ group were also an important factor in the activity. Electron- withdrawing groups exhibited better activity than electron-donating groups. Compounds **5a**, **5e** and **5g** showed better activity than **5f**. Compounds where the R^2^ group was 3-trifluoromethoxy showed the best inhibitory effect.

**Table 4:** IC_50_ values of target compounds. 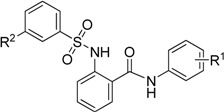

**Compd.**	**R^1^**	**R^2^**	**Inhibition (%) ^a^**	**IC_50_ (µM)**
**5a_2_**	3-chloro-4-fluoro	3-nitro	95.4 ± 3.3	1.32 ± 0.28
**5a_3_**	3-difluoromethoxy	3-nitro	89.0 ± 2.6	2.84 ± 0.52
**5a_4_**	3-trifluoromethoxy	3-nitro	83.8 ± 3.4	1.23 ± 0.12
**5a_8_**	2,4-dichloro	3-nitro	99.1 ± 2.3	0.37 ± 0.08
**5a_10_**	3-trifluoromethyl-4-methyl	3-nitro	90.7 ± 4.2	1.49 ± 0.09
**5g_1_**	2,4-dichloro	3-trifluoromethoxy	89.3 ± 3.3	0.91 ± 0.18
**5g_2_**	3-chloro-4-fluoro	3-trifluoromethoxy	98.6 ± 2.6	0.11 ± 0.05
**5g_3_**	3-trifluoromethyl-4-methyl	3-trifluoromethoxy	91.8 ± 3.5	0.83 ± 0.11
**5g_4_**	3-trifluoromethoxy	3-trifluoromethoxy	88.1 ± 2.7	1.03 ± 0.16
**5g_5_**	3,4-dichloro	3-trifluoromethoxy	80.8 ± 4.3	1.22 ± 0.20
**S-1647**	3,4-dichloro	3-nitro	79.3 ± 5.2	1.52 ± 0.12

^a^ Values represent the percent inhibition of ASBT at 10 µM of the test compounds and are the average of three independent experiments.

Finally, the compounds with inhibition rate above 80% were further assessed based on IC_50_ values and the results are listed in Table 4. All of the tested compounds showed considerable activity (IC_50_: 0.11–2.84 μM). Among of them, **5a_8_**, **5g_1_**, **5g_2_** and **5g_3_** were more active than the lead compound S-1647. Particularly, compound **5g_2_** which had the best inhibitory activity (IC_50_: 0.11 μM) was found to be 14- fold more active than S-1647 (IC_50_: 1.52 μM).

## 3. Experimental

### 3.1. General

All melting points were obtained on a Büchi Melting Point B-540 apparatus (Büchi Labortechnik, Flawil, Switzerland) and are uncorrected. Mass spectra (MS) were taken in ESI mode on an Agilent 1100 LC-MS system (Agilent, Palo Alto, CA, USA). Nuclear magnetic resonance spectroscopy was performed using a 400 MHz Bruker ARX-400 spectrometers (Bruker Bioscience, Billerica, MA, USA) with DMSO-*d*_6_ as solvent and TMS as an internal standard. All the starting materials were obtained from commercially available sources and used without further purification, unless otherwise specified. Yields were not optimized.

*Methyl 2-(3-Nitrophenylsulfonamido)benzoate* (**3a**). To a solution of **1a** (5.0 g, 21.4 mmol) in THF (60 mL) was added methyl 2-aminobenzoate (**2a**, 2.7 mL, 21.4 mmol) and then pyridine (1.7 mL, 21.4 mmol). The reaction mixture was stirred for 9 h at room temperature and then concentrated. To the residue was added water and then 5% HCl. The mixture was stirred for 0.5h and filtered. The filter cake was washed with water, dried and gave **3a** as a red solid (74.0% yield); m.p.: 127.1–128.0 °C. ^1^H-NMR δ: 3.75 (3H, s), 7.25 (1H, t, *J* = 6.0 Hz), 7.39 (1H, d, *J* = 6.0 Hz), 7.57 (1H, t, *J* = 6.0 Hz), 7.79 (1H, dd, *J_1_* = 6.0 Hz, *J_2_* = 1.2 Hz), 7.85 (1H, t, *J* = 6.4 Hz), 8.15 (1H, d, *J* = 6.4 Hz), 8.44–8.47 (2H, m), 10.49 (1H, s). MS *m/z*: 359.07 [M+Na]^+^.

*Methyl 3-(3-Nitrophenylsulfonamido)benzoate* (**3b**). Compound **3b** was obtained as a white solid (78.7% yield) from compounds **1a** and **2b** as described for **3a**; m.p.: 153.5–154.4 °C. ^1^H-NMR δ: 3.82 (3H, s) , 7.37–7.45 (2H, m), 7.66 (1H, d, *J* = 7.6 Hz), 7.72 (1H, s), 7.86 (1H, t, *J* = 8.0 Hz), 8.13 (1H, d, *J* = 7.6 Hz), 8.45 (1H, dd, *J_1_* = 8.0 Hz, J2= 2.0 Hz), 8.49 (1H, t, *J* = 2.0 Hz), 10.80 (1H, s). MS *m/z*: 335.16 [M-H]^−^.

*Methyl 4-(3-Nitrophenylsulfonamido)benzoate* (**3c**). Compound **3c** was obtained as a white solid (76.3% yield) from compounds **1a** and **2c** as described for **3a**; m.p.: 209.9–211.0 °C. ^1^H-NMR δ: 3.78 (3H, s), 7.25 (2H, d, *J* = 8.8 Hz), 7.84–7.89 (3H, m), 8.20 (1H, d, *J* = 8.0 Hz), 8.45 (1H, dd, *J_1_* = 8.0 Hz, J2= 2.0 Hz), 8.54 (1H, t, *J* = 2.0 Hz), 11.10 (1H, s). MS *m/z*: 335.16 [M−H]^−^.

*Methyl 2-(4-Nitrophenylsulfonamido)benzoate* (**3d**). Compound **3d** was obtained as a yellow solid (67.7% yield) from compounds **1b** and **2a** as described for **3a**; m.p.: 154.9–155.7 °C. ^1^H-NMR δ: 3.76 (3H, s), 7.25 (1H, t, *J* = 6.0 Hz), 7.40 (1H, d, *J* = 6.8 Hz), 7.58 (1H, t, *J* = 6.0 Hz), 7.80 (1H, dd, *J_1_* = 6.4 Hz, *J_2_* = 0.8 Hz), 8.01 (2H, d, *J* = 7.2 Hz), 8.35 (2H, d, *J* = 7.2 Hz), 10.55 (1H, s). MS *m/z*: 335.08 [M−H]^−^.

*Methyl 2-(3-Fluorophenylsulfonamido)benzoate* (**3e**). Compound **3e** was obtained as a white solid (72.6% yield) from compounds **1c** and **2a** as described for **3a**; m.p.: 122.3–123.5 °C. ^1^H-NMR δ: 3.80 (3H, s), 7.22 (1H, t, *J* = 8.0 Hz), 7.43 (1H, d, *J* = 8.0 Hz), 7.51-7.63 (5H, m), 7.83 (1H, dd, *J_1_* = 8.0 Hz, *J_2_* = 1.6 Hz), 10.42 (1H, s). MS *m/z*: 310.95 [M+H]+.

*Methyl 2-(3-Methoxyphenylsulfonamido)benzoate* (**3f**). Compound **3f** was obtained as a white solid (73.3% yield) from compounds **1d** and **2a** as described for **3a**; m.p.: 123.7–124.9 °C. ^1^H-NMR δ: 3.76 (3H, s), 3.81 (3H, s), 7.17–7.24 (3H, m), 7.34 (1H, d, *J* = 7.6 Hz), 7.47 (2H, t, *J* = 8.0 Hz), 7.58 (1H, t, *J* = 8.0 Hz), 7.84 (1H, dd, *J_1_* = 8.0 Hz, *J_2_* = 1.6 Hz), 10.37 (1H, s). MS *m/z*: 320.05 [M−H]^−^.

*Methyl 2-(3-(Trifluoromethoxy)phenylsulfonamido)benzoate* (**3g**). To a solution of **1e** (8.6 g, 33.1 mmol) in THF (60 mL) was added methyl 2-aminobenzoate (**2a**, 5.0 g, 33.1 mmol) and then pyridine (3.2 g, 39.7 mmol). The reaction mixture was stirred for 9 h at room temperature. To the residue was added water and then 5% HCl. The mixture was stirred for 0.5 h and extracted with dichloromethane. The dichloromethane layer was dried over MgSO4, concentrated in vacuo to afford **3g** as a red liquid (75.2% yield) that was used directly for the next reaction without further purification.

*2-(3-Nitrophenylsulfonamido)benzoic acid* (**4a**). To a solution of **3a** (5.7 g, 17.0 mmol) in the ethanol (20 mL) was added 10% aqueous sodium hydroxide (12 mL). The mixture was heated to 80 °C for 8 h and cooled to room temperature. The solution was concentrated and dissolved in water (50 mL). The mixture was adjusted to pH 2 with 6 N hydrochloric acid to give a white precipitate. The precipitate was filtered and washed with water to pH 7. The filter cake was dried to give **4a** (86.2% yield) as a white solid; m.p.: 219.8–220.5 °C. ^1^H-NMR δ: 7.17 (1H, t, *J* = 7.6 Hz), 7.47 (1H, d, *J* = 8.0 Hz), 7.56 (1H, t, *J* = 8.4 Hz), 7.82–7.88 (2H, m), 8.19 (1H, d, *J* = 7.6 Hz), 8.45–8.47 (2H, m), 11.28 (1H, s). MS *m/z*: 321.08 [M−H]^−^.

*3-(3-Nitrophenylsulfonamido)benzoic acid* (**4b**). Obtained as a white solid (86.1% yield) from compound **3b** as described for **4a**; m.p.: 249.0–250.2 °C. ^1^H-NMR δ: 7.34–7.41 (2H, m), 7.63 (1H, d, *J* = 7.2 Hz), 7.69 (1H, s), 7.86 (1H, t, *J* = 8.0 Hz), 8.12 (1H, d, *J* = 8.0 Hz), 8.45 (1H, dd, *J_1_* = 8.0 Hz, *J_2_* = 2.0 Hz,), 8.49 (1H, t, *J* = 2.0 Hz), 10.80 (1H, s), 13.10 (1H, s). MS *m/z*: 321.07 [M−H]^−^.

*4-(3-Nitrophenylsulfonamido)benzoic acid* (**4c**). Obtained as a white solid (83.9% yield) from compound **3c** as described for **4a**; m.p.: 269.4–270.6 °C. ^1^H-NMR δ: 7.22 (2H, d, *J* = 8.8 Hz), 7.81–7.89 (3H, m), 8.20 (1H, d, *J* = 8.0 Hz), 8.45 (1H, dd, *J_1_* = 8.0 Hz, J2= 2.0 Hz), 8.54 (1H, t, *J* = 2.0 Hz), 11.06 (1H, s), 12.80 (1H, s). MS *m/z*: 321.08 [M−H]^−^.

*2-(4-Nitrophenylsulfonamido)benzoic acid* (**4d**). Obtained as a yellow solid (82.5% yield) from compound **3d** as described for **4a**; m.p.: 238.1–239.2 °C. ^1^H-NMR δ: 7.16 (1H, t, *J* = 6.0 Hz), 7.47 (1H, d, *J* = 6.4 Hz), 7.55 (1H, t, *J* = 6.8 Hz), 7.89 (1H, dd, *J_1_* = 6.0 Hz, *J_2_* = 1.2 Hz), 8.05 (2H, d, *J* = 7.2 Hz), 8.34 (2H, d, *J* = 7.2 Hz), 10.39 (1H, s). MS *m/z*: 321.00 [M−H]^−^.

*2-(3-Fluorophenylsulfonamido)benzoic acid* (**4e**). Obtained as a white solid (88.3% yield) from compound **3e** as described for **4a**; m.p.: 187.1–188.2 °C. ^1^H-NMR δ: 7.16 (1H, t, *J* = 8.0 Hz), 7.48 (1H, d, *J* = 8.0 Hz), 7.51–7.66 (5H, m), 7.90 (1H, dd, *J_1_* = 8.0 Hz, *J_2_* = 1.6 Hz), 11.22 (1H, s). MS *m/z*: 294.09 [M−H]^−^.

*2-(3-Methoxyphenylsulfonamido)benzoic acid* (**4f**). Obtained as a white solid (82.7% yield) from compound **3f** as described for **4a**; m.p.: 157.1–158.3 °C. ^1^H-NMR δ: 3.76 (3H, s), 7.12 (1H, t, *J* = 8.0 Hz), 7.20 (1H, dd, *J_1_* = 8.0 Hz, *J_2_* = 2.4 Hz), 7.26 (1H, t, *J* = 2.0 Hz), 7.35 (1H, d, *J* = 8.0 Hz), 7.47 (1H, t, *J* = 8.0 Hz), 7.52-7.58 (2H, m), 7.89 (1H, dd, *J_1_* = 8.0 Hz, *J_2_* = 1.6 Hz), 11.08 (1H, s), 13.97 (1H, s). MS *m/z*: 306.12 [M−H]^−^.

*2-(3-(Trifluoromethoxy)phenylsulfonamido)benzoic acid* (**4g**). Obtained as a white solid (85.1% yield) from compound **3g** as described for **4a**; m.p.: 135.0–136.2 °C. ^1^H-NMR δ: 7.17 (1H, t, *J* = 8.0 Hz), 7.50 (1H, d, *J* = 8.0 Hz), 7.57 (1H, t, *J* = 8.0 Hz), 7.67-7.74 (3H, m), 7.84 (1H, d, *J* = 7.6 Hz), 7.89 (1H, dd, *J_1_* = 7.6 Hz, *J_2_* = 1.2 Hz), 11.17 (1H, s). MS *m/z*: 360.11 [M−H]^−^.

### 3.2. General Procedure for the Synthesis of Arylsulfonylaminobenzanilides

To a solution of **4a**–**g** (1 mmol) in dry DMF (10 mL) was added HOBt (1.5 mmol) and EDC**^.^**HCl (1.5 mmol). The reaction mixture was stirred at room temperature for 2 h, and then the substituted arylamines (2.0 mmol) and DIEA (2.0 mmol) were added, and then stirred at room temperature for 12 h, poured into ice-cold water. The precipitate was filtered, washed with water, and then recrystallized with ethyl acetate or purified by column chromatography (silica gel) to give the title compounds.

*N-(3,4-Dichlorophenyl)-2-(3-nitrophenylsulfonamido)benzamide* (**5a**_1_). White solid, 76.7% yield, m.p.: 185.0–186.3 °C. ^1^H-NMR δ: 7.30–7.34 (2H, m), 7.48–7.51 (2H, m), 7.57–7.62 (2H, m), 7.73 (1H, t, *J* = 8.0 Hz), 7.95 (1H, s), 8.07 (1H, d, *J* = 7.6 Hz), 8.32 (1H, d, *J* = 8.0 Hz), 8.39 (1H, s), 10.34 (1H, s), 10.41 (1H, s). MS *m/z*: 464.18 [M−H]^−^.

*N-(3-Chloro-4-fluorophenyl)-2-(3-nitrophenylsulfonamido)benzamide* (**5a**_2_). White solid, 80.6% yield, m.p.: 203.4–204.1 °C. ^1^H-NMR δ: 7.33–7.40 (3H, m), 7.45–7.53 (2H, m), 7.61 (1H, d, *J* = 7.2 Hz), 7.72 (1H, t, J =8.0 Hz), 7.87 (1H, dd, *J_1_* = 6.8 Hz, *J_2_* = 2.4 Hz), 8.06 (1H, d, *J* = 8.4 Hz), 8.32 (1H, dd, *J_1_* = 8.4 Hz, *J_2_* = 1.6 Hz), 8.38 (1H, t, J =4.0 Hz), 10.35 (1H, s), 10.38 (1H, s). MS *m/z*: 448.07 [M−H]^−^.

*N-(3-(Difluoromethoxy)phenyl)-2-(3-nitrophenylsulfonamido)benzamide* (**5a**_3_). Yellow solid, 43.3% yield, m.p.: 127.4–128.2 °C. ^1^H-NMR δ: 6.91 (1H, d, *J* = 8.0 Hz), 7.19 (1H, s), 7.31–7.42 (4H, m), 7.49–7.53 (2H, m), 7.63 (1H, d, *J* = 7.6 Hz), 7.71 (1H, t, *J* = 8.0 Hz), 8.08 (1H, d, *J* = 8.0 Hz), 8.31 (1H, dd, *J_1_* = 8.0 Hz, *J_2_* = 1.2 Hz), 8.38 (1H, t, *J* = 2.0 Hz), 10.33 (1H, s), 10.41 (1H, s). MS *m/z*: 462.15 [M−H]^−^.

*2-(3-Nitrophenylsulfonamido)-N-(3-(trifluoromethoxy)phenyl)benzamide* (**5a**_4_). White solid, 47.6% yield, m.p.: 141.4–142.0 °C. ^1^H-NMR δ: 7.08 (1H, d, *J* = 8.0 Hz), 7.33 (2H, t, *J* = 8.0 Hz), 7.44 (1H, t, *J* = 8.0 Hz), 7.49–7.54 (2H, m), 7.63 (1H, d, *J* = 7.6 Hz), 7.70 (2H, t, *J* = 8.0 Hz), 8.07 (1H, d, *J* = 7.6 Hz), 8.31 (1H, dd, *J_1_* = 8.4 Hz, *J_2_* = 1.6 Hz), 8.39 (1H, t, *J* = 2.0 Hz), 10.37 (1H, s), 10.42 (1H, s). MS *m/z*: 504.12 [M+Na]^+^.

*N-(3,5-Difluorophenyl)-2-(3-nitrophenylsulfonamido)benzamide* (**5a**_5_). White solid, 78.0% yield, m.p.: 181.1–181.9 °C. ^1^H-NMR δ: 7.01 (1H, d, *J* = 6.0 Hz), 7.23 (1H, t, *J* = 6.0 Hz), 7.35–7.41 (3H, m), 7.44–7.46 (1H, m), 7.48–7.52 (2H, m), 8.03 (1H, t, *J* = 6.4 Hz), 8.45 (1H, d, *J* = 6.4 Hz), 8.67 (1H, dd, *J_1_* = 6.8 Hz, *J_2_* = 1.2 Hz), 8.73 (1H, s), 10.24 (1H, s). MS *m/z*: 432.12 [M−H]^−^.

*N-(3-Hydroxy-4-methoxyphenyl)-2-(3-nitrophenylsulfonamido)benzamide* (**5a**_6_). Yellow solid, 65.1% yield, m.p.: 168.9–170.1 °C. ^1^H-NMR δ: 3.30 (3H, s), 7.01 (1H, d, *J* = 6.0 Hz), 7.23 (1H, t, *J* = 6.0 Hz), 7.35–7.40 (3H, m), 7.44–7.47 (1H, m), 7.49–7.52 (2H, m), 8.03 (1H, t, *J* = 6.4 Hz), 8.45 (1H, d, *J* = 6.4 Hz), 8.67 (1H, d *J* = 6.4 Hz), 8.73 (1H, s), 10.24 (1H, s). MS *m/z*: 442.08 [M−H]^−^.

*N-(3,5-Dichlorophenyl)-2-(3-nitrophenylsulfonamido)benzamide* (**5a**_7_). White solid, 72.3% yield, m.p.: 209.9–210.4 °C. ^1^H-NMR δ: 7.30–7.36 (3H, m), 7.51 (1H, t, *J* = 8.0 Hz), 7.60 (1H, d, *J* = 7.2 Hz), 7.64 (2H, d, *J* = 2.0 Hz), 7.73 (1H, t, *J* = 8.0 Hz), 8.06 (1H, d, *J* = 8.4 Hz), 8.31-8.33 (1H, m), 8.40-8.41 (1H, m), 10.31 (1H, s), 10.45 (1H, s). MS *m/z*: 464.07 [M−H]^−^.

*N-(2,4-Dichlorophenyl)-2-(3-nitrophenylsulfonamido)benzamide* (**5a**_8_). White solid, 49.6% yield, m.p.: 203.6–204.1 °C. ^1^H-NMR δ: 7.30–7.35 (2H, m), 7.49–7.59 (3H,m), 7.72 (1H, d, *J* = 2.0 Hz),7.83 (1H, t, *J* = 8.0 Hz), 7.93 (1H, d, *J* = 8.0 Hz), 8.14 (1H, dd, *J_1_* = 8.0 Hz, *J_2_* = 2.0 Hz), 8.38 (1H, t, *J* = 2.0 Hz), 8.45 (1H, d, *J* = 8.0 Hz), 10.24 (1H, s), 10.88 (1H, s). MS *m/z*: 464.10 [M−H]^−^.

*N-(3-Chlorophenyl)-2-(3-nitrophenylsulfonamido)benzamide* (**5a**_9_). White solid, 70.2% yield, m.p.: 167.3–168.1 °C. ^1^H-NMR δ: 7.15 (1H, d, *J* = 8.0 Hz), 7.31–7.35 (3H, m), 7.46 (1H, d, *J* = 8.4 Hz), 7.50 (1H, t, *J* = 8.0 Hz), 7.63 (1H, d, *J* = 7.6 Hz), 7.72 (1H, t, *J* = 8.0 Hz), 7.77 (1H, s), 8.07 (1H, d, *J* = 8.0 Hz), 8.32 (1H, dd, *J_1_* = 8.0 Hz, *J_2_* = 1.6 Hz), 8.39 (1H, t, *J* = 2.4 Hz), 10.33 (1H, s), 10.40 (1H, s). MS *m/z*: 430.09 [M−H]^−^.

*N-(4-Methyl-3-(trifluoromethyl)phenyl)-2-(3-nitrophenylsulfonamido)benzamide* (**5a**_10_). White solid, 40.3% yield, m.p.: 166.4–167.4 °C ^1^H-NMR δ: 2.40 (3H, s), 7.31–7.38 (3H, m), 7.51 (1H, t, *J* = 7.6 Hz), 7.64 (1H, d, *J* = 7.6 Hz), 7.70 (2H, t, *J* = 8.0 Hz), 7.97 (1H, d, *J* = 1.2 Hz), 8.06 (1H, d, *J* = 8.0 Hz), 8.27–8.30 (1H, m), 8.37 (1H, t, *J* = 2.0 Hz), 10.36 (1H, s), 10.42 (1H, s). MS *m/z*: 478.10 [M−H]^−^.

*N-(3-Chloro-4-fluorophenyl)-3-(3-nitrophenylsulfonamido)benzamide* (**5b**_1_). White solid, 55.3% yield, m.p.: 161.3–162.6 °C. ^1^H-NMR δ: 7.31–7.33 (1H, m), 7.37–7.45 (2H, m), 7.63–7.67 (3H, m), 7.86 (1H, t, *J* = 8.0 Hz), 8.01 (1H, dd, *J_1_* = 7.2 Hz, J2= 2.8 Hz), 8.13–8.15 (1H, m), 8.43–8.46 (1H, m), 8.51 (1H, t, *J* = 2.0 Hz), 10.41 (1H, s), 10.80 (1H, s). MS *m/z*: 448.75 [M−H]^−^.

*N-(3-(Difluoromethoxy)phenyl)-3-(3-nitrophenylsulfonamido)benzamide* (**5b**_2_). White solid, 57.7% yield, m.p.: 159.8–160.6 °C. ^1^H-NMR δ: 6.90 (1H, d, *J* = 8.0 Hz), 7.19 (1H, s), 7.31–7.44 (3H, m), 7.59 (1H, d, *J* = 8.0 Hz), 7.66 (3H, d, *J* = 8.0 Hz), 7.85 (1H, t, *J* = 8.0 Hz), 8.15 (1H, d, *J* = 8.0 Hz), 8.45 (1H, d, *J* = 8.0 Hz), 8.51 (1H, s), 10.39 (1H, s), 10.80 (1H, s). MS *m/z*: 462.49 [M−H]^−^.

*N-(3,4-dichlorophenyl)-3-(3-nitrophenylsulfonamido)benzamide* (**5b**_3_). Yellow solid, 60.8% yield, m.p.: 192.5–193.7 °C. ^1^H-NMR δ: 7.32 (1H, d, *J* = 8.0 Hz), 7.43 (1H, t, *J* = 8.0 Hz), 7.59–7.70 (4H, m), 7.85 (1H, t, *J* = 8.0 Hz), 8.09 (1H, d, *J* = 2.4 Hz), 8.15 (1H, d, *J* = 8.0 Hz), 8.45 (1H, d, *J* = 8.0 Hz), 8.51 (1H, s), 10.48 (1H, s), 10.80 (1H, s). MS *m/z*: 464.91 [M−H]^−^.

*4-(3-Nitrophenylsulfonamido)-N-(3-(trifluoromethoxy)phenyl)benzamide* (**5c**_1_). White solid, 51.3% yield, m.p.: 177.3–178.2 °C. ^1^H-NMR δ: 7.09 (1H, d, *J* = 8.8 Hz), 7.26 (2H, d, *J* = 8.0 Hz), 7.46 (1H, t, *J* = 8.0 Hz), 7,71 (1H, d, *J* = 8.0 Hz), 7.85–7.91 (4H, m), 8.23 (1H, d, *J* = 8.0 Hz), 8.47 (1H, dd, *J_1_* = 8.0 Hz, J2= 2.0 Hz), 8.57 (1H, s), 10.37 (1H, s), 11.04 (1H, s). MS *m/z*: 480.57 [M−H]^−^.

*N-(3,4-Dichlorophenyl)-4-(3-nitrophenylsulfonamido)benzamide* (**5c**_2_). White solid, 57.8% yield, m.p.: 189.2–190.5 °C. ^1^H-NMR δ: 7.25 (2H, d, *J* = 8.0 Hz), 7.59 (1H, d, *J* = 8.8 Hz), 7.69 (1H, dd, *J_1_* = 8.8 Hz, J2= 2.4 Hz), 7.83–7.90 (3H, m), 8.09 (1H, d, *J* = 2.0 Hz), 8.22 (1H, d, *J* = 8.0 Hz), 8.47 (1H, dd, *J_1_* = 8.0 Hz, J2= 2.0 Hz), 8.56 (1H, s), 10.34 (1H, s), 11.04 (1H, s). MS *m/z*: 464.65 [M−H]^−^.

*N-(2,4-Dichlorophenyl)-2-(4-nitrophenylsulfonamido)benzamide* (**5d**_1_). White solid, 76.1% yield, m.p.: 220.6–221.3 °C. ^1^H-NMR δ: 7.31–7.37 (2H, m), 7.46–7.57 (3H, m), 7.72 (1H, d, *J* = 2.0 Hz), 7.97 (2H, d, *J* = 8.8 Hz), 8.33 (3H, m), 10.25 (1H, s), 10.96 (1H, s). MS *m/z*: 464.08 [M−H]^−^.

*N-(3-Chloro-4-fluorophenyl)-2-(4-nitrophenylsulfonamido)benzamide* (**5d**_2_). White solid, 77.5% yield, m.p.: 226.4–227.1 °C. ^1^H-NMR δ: 7.35–7.40 (3H, m), 7.48–7.54 (2H, m), 7.64 (1H, d, *J* = 7.2 Hz), 7.85 (1H, dd, *J_1_* = 7.2 Hz, *J_2_* = 2.4 Hz), 7.91 (2H, d, *J* = 8.8 Hz), 8.21 (2H, d, *J* = 8.8 Hz), 10.33 (1H, s), 10.39 (1H, s). MS *m/z*: 448.07 [M−H]^−^.

*N-(4-Methyl-3-(trifluoromethyl)phenyl)-2-(4-nitrophenylsulfonamido)benzamide* (**5d**_3_). White solid, 53.7% yield, m.p.: 198.8–199.6 °C. ^1^H-NMR δ: 2.41 (3H, s), 7.33–7.40 (3H, m), 7.52–7.56 (1H, m), 7.67 (1H, d, *J* = 7.6 Hz), 7.74 (1H, d, *J* = 7.6 Hz),7.90 (2H, d, *J* = 8.8 Hz), 7.95 (1H, s), 8.18 (2H, d, *J* = 8.8 Hz), 10.36 (1H, s), 10.43 (1H, s). MS *m/z*: 478.21 [M−H]^−^.

*N-(3-(Difluoromethoxy)phenyl)-2-(4-nitrophenylsulfonamido)benzamide* (**5d**_4_). White solid, 69.1% yield, m.p.: 203.9–205 °C. ^1^H-NMR δ: 6.91 (1H, d, *J* = 8.8 Hz), 7.19 (1H, s), 7.33–7.41 (3H, m), 7.49–7.54 (2H, m), 7.65 (1H, d, *J* = 7.2 Hz), 7.92 (2H, d, *J* = 8.8 Hz), 8.21 (2H, d, *J* = 8.8 Hz), 10.33 (1H, s), 10.44 (1H, s). MS *m/z*: 462.15 [M−H]^−^.

*2-(4-Nitrophenylsulfonamido)-N-(3-(trifluoromethoxy)phenyl)benzamide* (**5d**_5_). White solid, 70.3% yield, m.p.: 198.8–199.4 °C. ^1^H-NMR δ: 7.08 (1H, d, *J* = 7.6 Hz), 7.33 (2H, d, *J* = 8.0 Hz), 7.44 (1H, t, *J* = 8.0 Hz), 7.53 (2H, d, *J* = 8.0 Hz), 7.65 (1H, d, *J* = 7.2 Hz), 7.73 (1H,s), 7.91 (2H, d, *J* = 8.8 Hz), 8.20 (2H, d, *J* = 8.8 Hz), 10.38 (1H, s), 10.40 (1H, s). MS *m/z*: 480.21 [M−H]^−^.

*N-(3,4-Dichlorophenyl)-2-(4-nitrophenylsulfonamido)benzamide* (**5d**_6_). White solid, 65.2% yield, m.p.: 243.4–243.6 °C. ^1^H-NMR δ: 7.32–7.34 (2H, m), 7.52 (1H, dd, *J_1_* = 8.4 Hz, *J_2_* = 2.4 Hz), 7.56 (2H, t, *J* = 8.8 Hz), 7.62 (1H, d, *J* = 7.2 Hz), 7.90 (2H, d, *J* = 8.8 Hz),7.92 (1H,s), 8.21 (2H, d, *J* = 8.8 Hz), 10.35 (1H, s), 10.39 (1H, s). MS *m/z*: 464.08 [M−H]^−^.

*N-(2,4-Dichlorophenyl)-2-(3-fluorophenylsulfonamido)benzamide* (**5e**_1_). White solid, 67.6% yield, m.p.: 146.3–147.6 °C. ^1^H-NMR δ: 7.29 (1H, t, *J* = 7.2 Hz), 7.40 (1H, d, *J* = 8.0 Hz), 7.50–7.64 (7H, m), 7.75 (1H, d, *J* = 2.0 Hz), 7.88 (1H, d, *J* = 8.0 Hz), 10.34 (1H, s), 10.90 (1H, s). MS *m/z*: 438.05 [M−H]^−^.

*N-(3-Chloro-4-fluorophenyl)-2-(3-fluorophenylsulfonamido)benzamide* (**5e**_2_). White solid, 52.1% yield, m.p.: 178.6–179.9 °C. ^1^H-NMR δ: 7.29 (1H, t, *J* = 7.6 Hz), 7.35 (1H, d, *J* = 8.0 Hz), 7.41–7.59 (7H, m), 7.70 (1H, d, *J* = 7.6 Hz), 7.95 (1H, dd, *J_1_* = 6.8 Hz, J2= 2.4 Hz), 10.41 (1H, s), 10.47 (1H, s). MS *m/z*: 421.28 [M−H]^−^.

*2-(3-Fluorophenylsulfonamido)-N-(4-methyl-3-(trifluoromethyl)phenyl)benzamide* (**5e**_3_). Yellow solid, 55.8% yield, m.p.: 168.8–169.6 °C. ^1^H-NMR δ: 2.41 (3H, s), 7.27 (1H, t, *J* = 7.3 Hz), 7.35 (1H, d, *J* = 8.4 Hz), 7.42 (2H, d, *J* = 8.1 Hz), 7.48–7.56 (3H, m), 7.72 (1H, d, *J* = 6.8 Hz), 7.78 (1H, d, *J* = 8.0 Hz), 8.03 (1H, s), 10.46 (1H, s), 10.48 (1H, s). MS *m/z*: 451.30 [M−H]^−^. 

*2-(3-Fluorophenylsulfonamido)-N-(3-(trifluoromethoxy)phenyl)benzamide* (**5e**_4_). White solid, 61.7% yield, m.p.: 166.8–168.7 °C. ^1^H-NMR δ: 7.11 (1H, d, *J* = 8.4 Hz), 7.26–7.34 (2H, m), 7.39–7.57 (6H, m), 7.62 (1H, d, *J* = 8.4 Hz), 7.70 (1H, d, *J* = 7.2 Hz), 7.79 (1H, s), 10.37 (1H, s), 10.52 (1H, s). MS *m/z*: 453.68 [M−H]^−^.

*N-(3,4-Dichlorophenyl)-2-(3-fluorophenylsulfonamido)benzamide* (**5e**_5_). White solid, 58.5% yield, m.p.: 179.1–180.6 °C. ^1^H-NMR δ: 7.27–7.33 (2H, m), 7.42–7.58 (5H, m), 7.60–7.64 (2H, m), 7.69 (1H, d, *J* = 8.0 Hz), 8.03 (1H, d, *J* = 1.6 Hz), 10.34 (1H, s), 10.53 (1H, s). MS-ESI *m/z*: 437.34 [M−H]^−^.

*N-(2,4-Dichlorophenyl)-2-(3-methoxyphenylsulfonamido)benzamide* (**5f**_1_). White solid, 51.5% yield, m.p.: 138.8–139.7 °C. ^1^H-NMR δ: 3.73 (3H, s), 7.19–7.30 (4H, m), 7.43–7.54 (4H, m), 7.61 (1H, d, *J* = 8.4 Hz), 7.75 (1H, d, *J* = 1.6 Hz), 7.88 (1H, d, *J* = 8.0 Hz), 10.35 (1H, s), 10.85 (1H, s). MS *m/z*: 449.44 [M−H]^−^.

*N-(3-Chloro-4-fluorophenyl)-2-(3-methoxyphenylsulfonamido)benzamide* (**5f**_2_). White solid, 57.8% yield, m.p.: 181.9–183.2 °C. ^1^H-NMR δ: 3.67 (3H, s), 7.12 (1H, dd, *J_1_* = 8.0 Hz, J2= 2.4 Hz), 7.25–7.29 (2H, m), 7.36–7.45 (3H, m), 7.51 (1H, t, *J* = 7.6 Hz), 7.56–7.60 (1H, m), 7.71 (1H, d, *J* = 7.6 Hz), 7.94 (1H, dd, *J_1_* = 6.8 Hz, J2= 2.4 Hz), 10.36 (1H, s), 10.46 (1H, s). MS *m/z*: 433.65 [M−H]^−^.

*2-(3-Methoxyphenylsulfonamido)-N-(4-methyl-3-(trifluoromethyl)phenyl)benzamide* (**5f**_3_).White solid, 56.2% yield, m.p.: 165.7–166.6 °C. ^1^H-NMR δ: 2.42 (3H, s), 3.63 (3H, s), 7.11 (1H, d, *J* = 8.0 Hz), 7.21 (1H, s), 7.26 (2H, t, *J* = 8.7 Hz), 7.35–7.44 (3H, m), 7.51 (1H, t, *J* = 7.7 Hz), 7.74 (1H, d, *J* = 7.7 Hz), 7.80 (1H, d, *J* = 8.0 Hz), 8.04 (1H, s), 10.42 (1H, s), 10.49 (1H, s). MS *m/z*: 463.58 [M−H]^−^.

*2-(3-Methoxyphenylsulfonamido)-N-(3-(trifluoromethoxy)phenyl)benzamide* (**5f**_4_). White solid, 63.5% yield, m.p.: 149.3–150.8 °C. ^1^H-NMR δ: 3.64 (3H, s), 7.09–7.27 (5H, m), 7.34–7.41 (2H, m), 7.46–7.53 (2H, m), 7.63 (1H, d, *J* = 7.6 Hz), 7.71 (1H, d, *J* = 7.6 Hz), 7.78 (1H, s), 10.33 (1H, s), 10.52 (1H, s). MS *m/z*: 465.22 [M−H]^−^.

*N-(3,4-Dichlorophenyl)-2-(3-methoxyphenylsulfonamido)benzamide* (**5f**_5_). White solid, 67.9% yield, m.p.: 160.3–161.4 °C. ^1^H-NMR δ: 3.66 (3H, s), 7.11 (1H, dd, *J_1_* = 8.4 Hz, J2= 2.4 Hz), 7.28–7.30 (3H, m), 7.35–7.39 (2H, m), 7.50 (1H, d, *J* = 8.0 Hz), 7.57–7.63 (2H, m), 7.69 (1H, d, *J* = 8.0 Hz), 8.01 (1H, d, *J* = 2.0 Hz), 10.27 (1H, s), 10.51 (1H, s). MS *m/z*: 449.57 [M−H]^−^.

*N-(2,4-Dichlorophenyl)-2-(3-(trifluoromethoxy)phenylsulfonamido)benzamide* (**5g**_1_). White solid, 69.6% yield, m.p.: 151.6–152.8 °C. ^1^H-NMR δ: 7.30 (1H, t, *J* = 7.6 Hz), 7.35 (1H, d, *J* = 8.0 Hz), 7.49–7.51 (2H, m), 7.62–7.78 (6H, m), 7.88 (1H, d, *J* = 7.6 Hz), 10.31 (1H, s),10.94 (1H, s). MS *m/z*: 503.12 [M−H]^−^.

*N-(3-Chloro-4-fluorophenyl)-2-(3-(trifluoromethoxy)phenylsulfonamido)benzamide* (**5g**_2_). White solid, 68.1% yield, m.p.: 157.3–158.6 °C. ^1^H-NMR δ: 7.28–7.34 (2H, m), 7.42 (1H, t, *J* = 8.8 Hz), 7.50 (1H, t, *J* = 8.0 Hz), 7.54–7.74 (6H, m), 7.96 (1H, dd, *J_1_* = 6.8 Hz, J2= 2.4 Hz), 10.46 (2H, s). MS *m/z*: 487.85 [M−H]^−^.

*N-(4-Methyl-3-(trifluoromethyl)phenyl)-2-(3-(trifluoromethoxy)phenylsulfonamido)benzamide* (**5g**_3_). White solid, 55.7% yield, m.p.: 179.4–180.5 °C. ^1^H-NMR δ: 2.42 (3H, s), 7.31 (1H, d, *J* = 7.6 Hz), 7.35 (1H, d, *J* = 8.4 Hz), 7.42 (1H, d, *J* = 8.4 Hz), 7.51 (1H, t, *J* = 8.0 Hz), 7.56–7.62 (2H, m), 7.64 (1H, s), 7.70–7.73 (2H, m), 7.77 (1H, d, *J* = 8.4 Hz), 8.06 (1H, s), 10.47 (1H, s), 10.51 (1H, s). MS *m/z*: 517.52 [M−H]^−^.

*N-(3-(Trifluoromethoxy)phenyl)-2-(3-(trifluoromethoxy)phenylsulfonamido)benzamide* (**5g**_4_). White solid, 62.3% yield, m.p.: 169.1–170.4 °C. ^1^H-NMR δ: 7.11 (1H, d, *J* = 8.4 Hz), 7.29–7.34 (2H, m), 7.46–7.51 (2H, m), 7.57–7.62 (3H, m), 7.66 (1H, s), 7.71 (2H, t, *J* = 7.2 Hz), 7.81 (1H, s), 10.43 (1H, s), 10.52 (1H, s). MS *m/z*: 519.12 [M−H]^−^.

*N-(3,4-Dichlorophenyl)-2-(3-(trifluoromethoxy)phenylsulfonamido)benzamide* (**5g**_5_). White solid, 66.7% yield, m.p.: 160.5–161.6 °C. ^1^H-NMR δ: 7.32 (2H, t, *J* = 8.0 Hz), 7.51 (1H, t, *J* = 8.0 Hz), 7.58–7.74 (7H, m), 8.05 (1H, d, *J* = 2.0 Hz), 10.40 (1H, s), 10.53 (1H, s). MS *m/z*: 503.75 [M−H]^−^.

### 3.3. ASBT Inhibition Assay

HEK293T was obtained from the American Type Culture Collection (Manassas, VA, USA), and grown in MEM supplemented with 100 U/mL penicillin and 100 mg/mL streptomycin and 10% heat-inactivated fetal bovine serum. Human ASBT expression construct was prepared as previously described [18]. HEK293T cells were seeded in 12 well plates and transiently transfected with 0.5 μg/well pcDNA3.1/ASBT or negative control plasmid pcDNA3.1 using lipofactamine 2000 (Invitrogen, Carlsbad, CA, USA) according to the manufacturer’s instructions. Twenty-four hours after transfection, HEK293T cells were assayed for taurocholic acid uptake as previously described with minor modifications [19]. Briefly, cells were washed twice with warm wash & uptake buffer (116 mM NaCl, 5.3 mM KCl, 1.1 mM KH_2_PO_4_, 0.8 mM MgSO_4_, 1.8 mM CaCl_2_, 11 mM D-dextrose/D-glucose, and 10 mM HEPES, pH 7.4), then cells were incubated with the same buffer containing the indicated concentrations of test compounds (dissolved in dimethyl sulfoxide) and 1 μCi/mL of [^3^H]-taurocholic acid(TCA, 0.2 μM) (PerkinElmer Life Sciences) for 10 min. To terminate the transport process, the plates were chilled on ice and the cells were immediately washed with ice-cold buffer three times. Cells were lysed with 0.3 mL lysis buffer (0.5% triton x-100) and shaken vigorously for 20 min. The radioactivity of the cell lysate was counted using a MicroBeta^2^ Liquid Scintillation and Luminescence Counter (PerkinElmer Life Sciences). Protein concentration of the lysate was used to normalize uptake activity.

The inhibition rate was calculated using the following formula:

Inhibition Rate (%) = [(1 − (A − C)/(B − C)] × 100

A, [^3^H] uptake value of test compound in [^3^H]-TCA buffer added to pcDNA3.1/ASBT transfected cells; B, [^3^H] uptake value of DMSO (without inhibitor) in [^3^H]-TCA buffer added to pcDNA3.1/ASBT transfected cells; C, [^3^H] uptake value of Blank sample (DMSO) in [^3^H]-TCA buffer added to empty vector (pcDNA3.1) transfected cells. The IC_50_ value was defined as the inhibitor concentration that gives 50% taurocholate uptake compared to the no inhibitor control and calculated using SigmaPlot 10.0 software.

## 4. Conclusions

A series of novel arylsulfonylaminobenzanilide derivatives were designed and synthesized. Their inhibitory activities against ASBT were assessed. In general, most of them had considerable ASBT inhibitory activity. In particular, four compounds (**5a_8_**, **5g_1,_5g_2_** and **5g_3_**) were superior to the lead compound S-1647, especially compound **5g_2_** which exhibited the most inhibitory effect on ASBT transport activity with an IC_50_ value of 0.11 μM, or 14-fold more potent than S-1647. It is not known if these compounds can pass the cell membrane using a transporter or permissively, so future studies will address this issue *in vitro* in cells and *in vivo* in animal models. It is unlikely that these compounds will have much cytotoxicity as both HEK293T and CACO2 cells showed no morphological differences when treated with 20 μM of the compounds for 24 h.
